# Searching for the social determinants of health: observations from evidence synthesis publications

**DOI:** 10.1186/s13643-024-02551-y

**Published:** 2024-05-16

**Authors:** Rosie Hanneke, Amelia Brunskill

**Affiliations:** Library of the Health Sciences, University of Illinois Chicago, 1750 W Polk St. MC 763, Chicago, IL 60612 USA

**Keywords:** Evidence synthesis, Review literature as topic, Systematic reviews, Information search and retrieval, Health equity, Social determinants of health, Evidence-based public health

## Abstract

**Background:**

The social determinants of health (SDOH) are the focus of an exponentially increasing number of publications, including evidence syntheses. However, there is not an established standard for searching for SDOH literature. This study seeks to identify published evidence syntheses pertaining to the SDOH, analyzing the search strategies used and the studies included within these reviews. The primary objectives are to compare search strategies and create a test set of SDOH publications.

**Methods:**

We searched PubMed, Embase, and Scopus for evidence syntheses that mentioned the SDOH in their research questions and included an SDOH search strategy. Relevant data extracted from each review included databases searched; search terms used for the SDOH; conceptual frameworks referenced; and the citations of primary studies included in the reviews, which were compiled to form a test set of cited papers. The relative recall of the respective search strategies was tested by documenting the total number of MEDLINE results each retrieved and the number of test set papers retrieved.

**Results:**

Sixty-four evidence syntheses were identified and included in the analysis, and 2750 cited papers were extracted. Findings indicate few commonalities across search strategies in search terms used, the total number of results retrieved, and the number of test set cited papers retrieved. One hundred and ninety-three unique MeSH terms and 1385 unique keywords and phrases were noted among the various search strategies. The number of total results retrieved by the SDOH search strategies ranged from 21,793 to over 16 million. The percentage of cited papers retrieved by the search strategies ranged from 2.46 to 97.9%. Less than 3% of the cited papers were indexed with the Social Determinants of Health MeSH.

**Conclusions:**

There has been little consistency across evidence syntheses in approaches to searching for SDOH literature. Differences in these strategies could have a significant impact on what literature is retrieved, included in reviews, and, consequently, incorporated into evidence-based practice. By documenting these differences and creating a set of papers relevant to SDOH, this research provides a snapshot of the current challenges in searching for SDOH content and lays the groundwork for the creation of a standardized search approach for SDOH literature.

**Supplementary Information:**

The online version contains supplementary material available at 10.1186/s13643-024-02551-y.

## Background

In recent years, health sciences researchers have turned their focus from individual health behaviors upstream to the social and structural determinants of health, seeking to understand larger, systemic causes of health disparities [1]. The social determinants of health (SDOH) are the focus of an exponentially increasing number of publications across health sciences disciplines in the twenty-first century [2, 3], including evidence syntheses. A few common frameworks are cited in Table 1, along with their respective definitions of SDOH.

**Table 1:** Common frameworks

Source and citation	Definition
WHO Commission on Social Determinants of Health [4]	“the circumstances in which people grow, live, work, and age, and the systems put in place to deal with illness. The conditions in which people live and die are, in turn, shaped by political, social, and economic forces.”
HealthyPeople 2030, U.S. Department of Health and Human Services, Office of Disease Prevention and Health Promotion [5]	“the conditions in the environments where people are born, live, learn, work, play, worship, and age that affect a wide range of health, functioning, and quality-of-life outcomes and risks. SDOH can be grouped into 5 domains: Economic Stability, Education Access and Quality, Health Care Access and Quality, Neighborhood and Built Environment, [and] Social and Community Context.”
Dahlgren and Whitehead, Determinants of Health Rainbow Model [6]	[The main determinants of health] “can be thought of as a series of layers, one on top of the other. Overall, there are the major structural environment. Then there are the material and social conditions in which people live and work…Mutual support from family, friends, neighbours and the local community comes next. Finally, there are actions taken by individuals.”

There has long been a call to incorporate considerations of health equity and upstream determinants into public health evidence syntheses such as systematic, scoping, and rapid reviews [7–11], given that failure to properly incorporate these factors can result in limited applicability of review findings [12, 13]. However, despite the significant literature pointing to the importance of considering equity and social determinants in reviews, there is a wide divergence in how these concepts are operationalized in review methods [14]. One guiding framework is PROGRESS and its expansion, PROGRESS-Plus [15], first published in 2014 by O’Neill and co-authors. Evidence synthesis authors are encouraged to incorporate this framework, “a list of factors associated with effects on equity,” in the design of systematic reviews and other evidence syntheses [16].

Parallel to the evolution of the SDOH conversation in health sciences research, health sciences librarians and other expert searchers have likewise turned their focus to the concepts of SDOH and health equity in recent years, particularly in the context of ongoing conversations about the importance of comprehensive search methods in evidence synthesis. In 2014, Sivan pointed to a need for systematic incorporation of SDOH terms into PubMed and other frequently used bibliographic databases [17]. This same year was the first that a Medical Subject Heading (MeSH) term for social determinants of health was introduced to MEDLINE indexing [18].

In order to capture a wider range of literature than may be identified by either subject headings or keywords alone, expert searchers frequently use search hedges or filters, both of which consist of a pre-selected combination of search terms for a specific database, to assist them in developing comprehensive search strategies. While PubMed once offered a comprehensive search query for the concept of *health disparities and minority health*, including some terms related to social determinants, this query is no longer updated as of 2019 [19]. Despite the near-ubiquity of the topic of SDOH in current health and library sciences conversations, there does not yet exist a validated search hedge or filter for identifying SDOH literature.

As described by Campbell, the terms filters and hedges are sometimes used interchangeably, but more traditionally filters refine a search to studies with specific characteristics, while hedges are focused on describing subject searches [20]. Under this definition, examples of filters constructed by researchers include ones pertaining to study type [21, 22], population [23, 24], and geography [25, 26], while examples of constructed hedges include ones for deprescribing [27], adverse effects of medical devices [28], acute kidney injury [29], patient-based benefit-risk assessment (BRA) of medicines [30], and public involvement in health efforts [31]. For other researchers to be able to reuse the developed strategy with an informed sense of its anticipated performance, it should be validated by formal testing and reporting of its relative recall and precision when tested against a gold standard set of relevant publications.

In a study by Prady and co-authors, who developed a search filter to identify studies related to the concept of equity [32], search terms from published, equity-focused reviews were sorted into categories of *concepts* (e.g., health equity, social determinants) and *measures* (e.g., poverty, education level). The authors ultimately recommended that researchers conducting evidence syntheses employ a “combined approach” using both specific equity terms and non-specific terms relating to how equity-focused reviews are often reported. One can assume a significant overlap between the literature identified by this equity filter and that concerned with the SDOH, and the authors seem to use the terms equity and SDOH interchangeably. However, there is a need for clear, evidence-based guidance on how to search for concepts and measures explicitly derived from an SDOH framework, especially as this framework has taken center stage in recent years.

The SDOH is not a single concept with a narrow or precisely defined scope. Conceptual frameworks describing the SDOH number in the dozens and vary widely in how they define the social determinants and describe relationships among them, at times offering conflicting definitions [33–37]. As a result of the myriad overlapping yet divergent definitions of the concept, it can be overwhelming to know where to begin in selecting search terms when conducting a review on the SDOH. Without an up-to-date search hedge or other standards for searching on the SDOH, review authors must build their own searches for this concept when designing search strategies for systematic, scoping, and other types of reviews.

This study seeks to examine published SDOH database search strategies from a representative sample of evidence syntheses. Its primary objective is to summarize the SDOH search strategies from these reviews and explore how this information might inform best practices for systematic searching of the SDOH literature. A secondary objective is to create a test set of SDOH papers by compiling publications included in the identified reviews to use for further analysis and compare the relative recall of the reviews' search strategies. Through pursuing these two objectives, we aim to lay the groundwork for creating a validated search hedge for this concept in the future.

## Methods

The work undertaken for this study had five primary components:

1. Identification of evidence syntheses that contain a search strategy for SDOH;
2. Analysis of the identified search strategies, including terms used, databases searched, and if the authors referenced a specific SDOH framework;
3. Extraction of included studies ("cited papers") from the evidence syntheses to form a potential test set of SDOH papers;
4. Analysis of cited papers test set to identify frequently-used terms and subject headings;
5. Comparison of SDOH search strategies for the number of results retrieved and recall of cited papers.

We searched PubMed, Embase, and Scopus on March 24, 2022, for a representative sample of systematic reviews, scoping reviews, and other evidence syntheses by searching social, determinant*, and *review* in the title fields. We also searched Cochrane Library for reviews with the terms *social* and determinant* in the title field. No restrictions were placed on the publication date. We exported these results to EndNote v20, removed duplicates, and imported the remaining records to Covidence. The primary investigator used Covidence to screen the titles and abstracts, then full-text papers, against the following inclusion criteria:

- Article is an evidence synthesis or evidence synthesis protocol that explicitly addresses the concept of SDOH, including but not limited to the following evidence synthesis types: systematic review, meta-analysis, scoping review, rapid review, integrative review, critical review;
- Review focuses on all aspects of health *or* an individual health outcome or behavior; e.g., “social determinants of physical activity,” “Social determinants of mental health,” “built environment as a social determinant of health”;
- Publication includes a full search strategy, including the *social determinants* concept, for at least one database, i.e., there is a list of terms used to search for social determinants.

### Analysis of search strategies

The search strategies of these reviews were extracted and analyzed using Microsoft Excel and Access. Relevant data extracted from each review included definitions or conceptual frameworks cited by review authors in the “Methods” section; databases searched; and search terms used for the SDOH, including keywords and controlled vocabulary (MeSH).

### Extraction of included studies (“cited papers”) from the evidence syntheses

For each of the evidence syntheses located, we extracted the citations of primary studies that had been chosen for inclusion within that review (these will henceforth be referred to as the test set of “cited papers”). In order to create a searchable block of these papers for further analysis, we searched for each study in PubMed, noting whether it appeared in that database. If it did, we recorded its PubMed ID number (PMID) in Microsoft Excel and combined these PMIDs to form the cited papers test set.

### Analysis of cited papers

We retrieved the MeSH term indexing for the cited papers test set by creating a search string for all identified PMIDs and running it in Ovid MEDLINE. This provided a count of all MeSH terms included within that set of records. In order to isolate the MeSH terms relevant to the social determinants of health, we removed the following from this list: check tags [38], terms related to geographic locations (e.g., “California”), investigative techniques (e.g., “logistic models”), and individual diseases or conditions (e.g., “diabetes mellitus”). After deleting these, we noted the remaining MeSH terms and grouped them by how frequently they were used among cited papers. We took the same approach to keywords (KW field), ordering them by frequency of use and removing ones using the same criteria as for the MeSH terms.

This set of PMIDs for the cited papers was also compared with the set of literature indexed with the MeSH term “social determinants of health.” Through this comparison, we ascertained how frequently this MeSH term was applied to the literature that researchers had identified as relevant to SDOH.

### Comparison of SDOH search strategies

In April 2023, we executed each SDOH search strategy for PubMed and Ovid MEDLINE, searching them in the platform indicated by the authors. We noted the number of records identified by each search.

We translated the PubMed search strategies to Ovid MEDLINE using the Polyglot Search Translator [39]. We then ran all search strategies, including those originally in Ovid MEDLINE format and those translated from PubMed to Ovid, against the set of PMIDs from the cited papers using Ovid MEDLINE. Ovid MEDLINE was used for all strategies, irrespective of the original platform used by the review authors, due to limitations with testing long PMID strings in PubMed. We noted the number of cited papers retrieved by each SDOH search strategy to analyze the respective recall of the strategies.

## Results

**Fig. 1:**
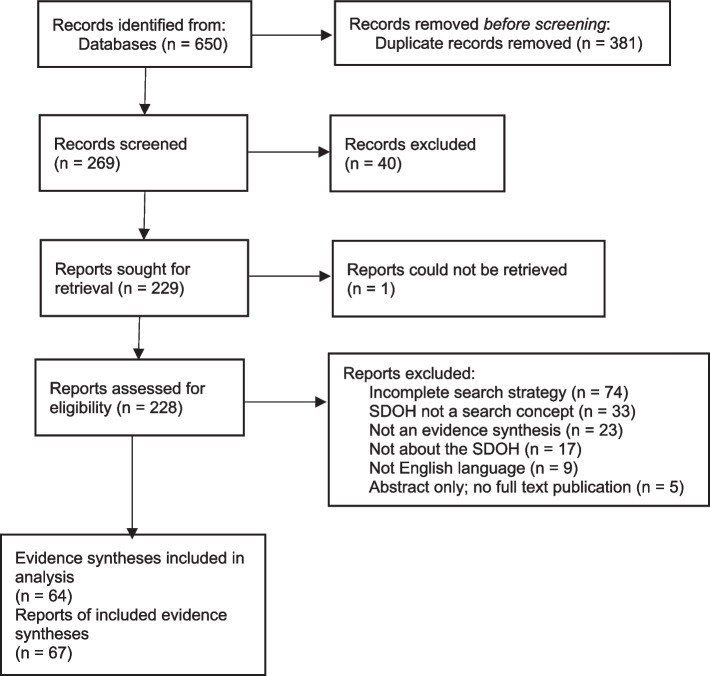
Flow diagram

Our search for evidence syntheses that contain a search strategy for SDOH in PubMed, Embase, and Scopus resulted in an initial set of 650 records, which we exported to EndNote. After removing 381 duplicates, the remaining 269 references were uploaded to Covidence (Fig. 1). Sixty-four reviews, described in 67 publications, were ultimately included in the analysis (complete list provided in the Additional  file 1).

### Definitions and conceptual frameworks

Review authors cited over 40 frameworks, models, definitions, and other publications (hereafter called “frameworks”) in their methods sections when describing the sources that guided their characterizations of the SDOH and/or their search term selection. Among the 64 reviews, 46 reviews cited at least one framework. Eighteen of these named multiple frameworks while 28 reviews cited a single framework.

The World Health Organization’s Commission on Social Determinants of Health was cited by 20 reviews, making it the most-used source. This framework, written by Solar and Irwin and published in 2008 [4] was later published in a 2010 version [40]; alternatively, some reviews cited a summarized version from *The Lancet* [41]*.* Eleven reviews cited the HealthyPeople 2020 SDOH framework [42], and two reviews cited the more recent HealthyPeople 2030 [5]. Eight reviews cited PROGRESS [16] and/or PROGRESS-Plus [15]. All other frameworks were cited either one or two times.

Sixteen reviews explicitly mentioned using these frameworks to guide the selection of search terms for their review. For example, Baker et al. reported that “General SDH and [Health Inequities] terms were identified from the WHO’s Commission on the Social Determinants of Health report and further clarified from glossaries on social epidemiology and health inequalities [43].” The other reviews cited frameworks generally, without specifying exactly the steps to which they were applied, or cited them in relation to another aspect of the study design, such as inclusion criteria or data extraction.

### Databases searched

Across all the reviews, over 50 different databases were searched (Table 2). An exact count of databases could not be determined due to inconsistencies and imprecisions in the way authors described their database searches. In particular, the frequent reporting error of listing a vendor platform rather than the specific databases searched within it (e.g., “ProQuest”) prevents this analysis. Among the 64 reviews, 62 searched either PubMed, Ovid MEDLINE, or both PubMed and Ovid MEDLINE, or indicated that they searched MEDLINE without specifying the platform. The databases reported as “Web of Science” and “Cochrane” reflect several possible resources searched under these products. The Clarivate Web of Science platform may search different date ranges and products including citation indexes, MEDLINE, and other databases, depending on the searcher's institutional subscriptions and backfile purchases [44]. Cochrane Library also searches multiple collections, including the Cochrane Database of Systematic Reviews (CDSR), Cochrane Central Register of Controlled Trials (CENTRAL), Cochrane Clinical Answers, and records from external (non-Cochrane-produced) resources [45]. The majority of reviews did not report which collections were searched within these two vendors; we have provided additional detail when it was given by the authors.

**Table 2:** Databases searched in at least 10 reviews

Database	Count
MEDLINE (all platforms)	62
PubMed only	42
Ovid MEDLINE only	10
Both PubMed and Ovid MEDLINE	4
MEDLINE (no platform specified)	6
Embase (all platforms)	28
CINAHL (all platforms)	27
Scopus	21
Web of Science (all versions)	21
No database or collection specified	17
WOS Core Collection, exact coverage not specified	2
Multiple individual WOS collections specified (e.g., SSCI, A&HCI)	2
APA PsycINFO (all platforms)	15
Cochrane (all versions)	15
No database or collection specified	7
CENTRAL	7
CDSR	1

The 62 reviews whose searches included a PubMed, Ovid MEDLINE, or generic MEDLINE search strategy were collected as the core dataset to form search term analysis. One of these 62 was excluded due to the published searched strategy containing extensive additional terms that had been added by PubMed’s Automatic Term Mapping (ATM) feature; these additional terms prevented straightforward analysis of the authors’ intended search methods. The following analyses were therefore conducted on the dataset of search strategies extracted from the remaining 61 reviews.

### MeSH terms

Thirty-nine of the 61 reviews (63.9%) used MeSH terms; to be precise, these 39 search strategies explicitly indicated that the terms were searched in the MeSH field. These search strategies used anywhere from 1 MeSH term to 46 of them (median = 12, *M* = 13.51). Seven terms indicated by authors to be MeSH, upon further review, were found to be neither current nor previous iterations of MeSH terms. In total, there were 193 unique MeSH terms, and one floating subheading (education), used to search for the SDOH concept. The two most commonly appearing MeSH terms (Table 3) were social determinants of health (in use since 2014) and socioeconomic factors (in use since 1968). Each was used in 28 of the search strategies. A full list of all MeSH terms used to search for the SDOH concept is available in the Additional file 1.

**Table 3:** MeSH terms appearing in at least 10 reviews

MeSH term	Count
Social determinants of health	28
Socioeconomic factors	28
Health status disparities	16
Educational status	12
Health services accessibility	11
Healthcare disparities	11
Social class	11
Residence characteristics	10
Unemployment	10
Vulnerable populations	10

### Keywords, title, and abstract terms

Fifty-eight of the 61 search strategies incorporated keywords, i.e., non-controlled vocabulary terms. The number of keywords used in each of these reviews ranged from one to 218 (median = 20, *M* = 38.75), and there was a total of 1385 unique keywords and phrases used across all 58 reviews. The remaining three reviews used MeSH terms exclusively and did not search other fields such as title or abstract. Keywords that were variations of a single term (e.g., social determinants, social determinant, social determinant* with truncation) were counted individually rather than grouped together. The keywords used most frequently (Table 4) included a mix of both terms describing general concepts (*social determinants, socioeconomic status*) and specific keywords for individual determinants (*income, race, unemployment*). A full list of all keywords and phrases used to search for the SDOH concept is available in the Additional file 1.

**Table 4:** Keywords appearing in at least 10 reviews

Keyword	Count
Poverty	30
Social determinants of health	18
Income	17
Social determinants	16
Social support	15
Social class	13
Socioeconomic status	13
Gender	12
Race	12
Social determinant* (truncated)	11
Socioeconomic	11
Employment	10
Rural	10
Socioeconomic factors	10
Unemployment	10

Most of the reviews incorporated at least one advanced search technique into their keyword searches. Thirty-nine reviews used at least one instance of truncation or wildcard searching among their keywords. A minority of reviews (*n* = 23) used either double quotation marks or hyphens to search two or more keywords together as a bound phrase. Ten of the 13 reviews that searched Ovid MEDLINE also incorporated proximity/adjacency operators into their keyword searches.

### Number of results

**Fig. 2:**
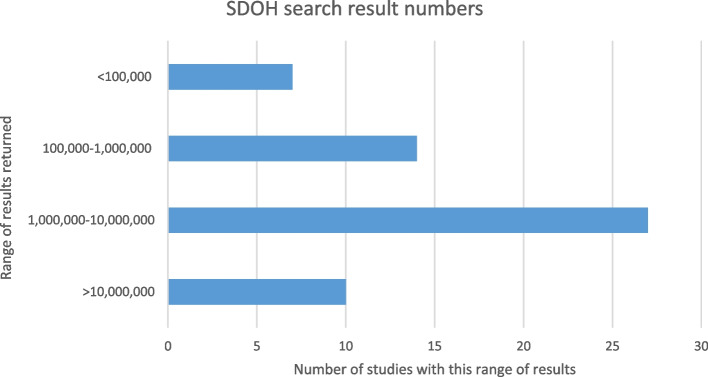
SDOH search result numbers

The SDOH portion of each review's search strategy was run in either PubMed or Ovid MEDLINE, according to which one the authors had stated they searched. We searched first for the SDOH-related terms on their own, independent of additional search concepts. An additional three search strategies were excluded at this stage, leaving 58 search strategies; because of changes to the MeSH terms used in these three reviews, it was not possible to execute the searches as they had been conducted at the time of the reviews. The number of results for these isolated SDOH search strategies ranged from 21,793 (Morone 2017) to 16,423,265 (Maness 2016). In Fig. 2, we cluster the search strategies by the number of results to highlight how they varied within those extremes, with a plurality retrieving between one million and ten million results.

### Cited papers

The original dataset of 64 reviews, i.e., the 61 reviews whose search strategies are described above together with the remaining three reviews, was revisited to extract data from the papers cited by each review. Twelve of the 64 reviews were excluded from this stage of analysis, either because they did not provide a clear list of citations for their included studies (*n* = 8) or because they were protocols and therefore did not yet have any studies selected for inclusion (*n* = 4).

The remaining 52 reviews reported a total of 2750 cited papers representing 2714 studies. Within this test set of 2750 cited papers, 2554 were found in PubMed and their PMIDs were noted. The other 196 cited papers are not found in PubMed.

Among the 2554 cited papers that were in PubMed, 36 duplicates were found, i.e., papers that had been cited in more than one of the evidence syntheses. After removing these, 2518 unique PMIDs remained. This set, referred to as the “cited papers” test set, represents 2518 unique publications in PubMed that have been selected by review authors as relevant to the topic of SDOH.

### MeSH terms from cited papers

When the set of 2518 cited papers was searched in PubMed in combination with the “social determinants of health” MeSH term, 2.42% (*n* = 61) were retrieved. There were 1401 cited papers with a date created (DA) prior to 2014 which could not have used the “social determinants of health” MeSH term, which was added to the MeSH database in 2014 [46]. Two cited papers had no DA field. When all remaining PMIDs, which had a Date Created between 2014 and 2022 (*n* = 1115), were searched in combination with “social determinants of health”[mesh], the percentage indexed with SDOH MeSH rose to 5.47%. “Socioeconomic Factors,” the other MeSH term which appeared most frequently among SDOH search strategies, retrieved 36.73% (*n* = 925) of the cited papers.

Cited papers were indexed with 38,393 MeSH terms. After deleting 10,918 check tags (human = 2374, male = 1461, female = 1724, adult = 1115, middle-aged = 882, adolescent = 773, aged = 637, young adult = 522, child = 392, aged, 80 and over = 255, pregnancy = 249, child, preschool = 191, infant = 169, infant, newborn = 167, animals = 7), 27,475 MeSH terms remained, including duplicates. Unique MeSH terms appearing among the cited papers totaled 2,482. There were 778 MeSH terms that appeared in the indexing for a single article, each appearing once. Each of the remaining 1704 MeSH terms appeared in the MeSH terms of two or more cited papers.

After removing MeSH terms related to geographic locations, investigative techniques, and individual diseases or conditions, the 20 most frequently appearing MeSH terms were collected (Table 5).

**Table 5:** Twenty most frequent MeSH terms among cited papers (excluding investigative techniques, locations, diseases, and conditions)

MeSH term	Count
Risk factors	546
Socioeconomic factors	493
Black or African American	221
Health services accessibility	190
Poverty	187
Healthcare disparities	183
Age factors	176
Sex factors	166
Health status disparities	155
White people	152
Emergency service, hospital	150
Residence characteristics	150
Social class	147
Health knowledge, attitudes, practice	146
Urban population	140
Educational status	138
Ethnicity	137
Veterans	130
Hispanic or Latino	129
Patient acceptance of health care	128

### Keywords from cited papers

Of the 2518 cited papers, 663 had keywords (listed in the KW field in MEDLINE), with a total of 3577 keywords total across the papers. These keywords were compiled, trimmed, and reviewed for frequency. After deleting terms that would have been check tags, geographic regions, diseases, conditions, and investigative techniques, the top 20 most frequent terms/phrases were identified which corresponded to those that appeared in twelve or more papers (see Table 6). For many of these, there were synonyms and variations on these terms that also appeared and sometimes the word appeared as part of a larger phrase. For example, in addition to race, there were other variations, some of which included: race and ethnic disparities, race and ethnicity, race factors, race/ethnicity, race-related stress, racial and ethnic disparities, racial bias, racial discrimination, racial disparities, racial disparity, racial health disparities, racial identity, racial misclassification, racial/ethnic differences, racial/ethnic differences in health and health care, racial/ethnic disparities, and racism. It is possible that other keywords might have been more common if all variations and synonyms were counted together.

**Table 6:** Twenty keywords appearing most frequently among cited papers (excluding investigative techniques, locations, diseases, and conditions)

Keyword	Count
Social determinants of health	39
Mental health	30
Health disparities	26
Socioeconomic status	25
Epidemiology	22
Risk factors	22
Emergency department	16
Race	16
Multimorbidity	15
Social support	15
Veterans	15
Disparities	14
Poverty	14
African Americans	13
Mortality	13
Prevalence	13
Screening	13
Ethnicity	12
Public health	12
Social determinants	12

### Retrieval of cited papers by collected search strategies

**Fig. 3:**
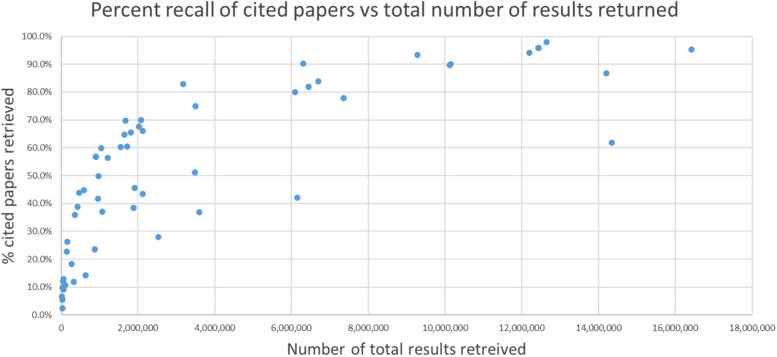
Percent recall of cited papers vs. total number of results returned

The percentage of the cited papers retrieved by the search strategies varied from a low of 2.46% to a high of 97.9%. Figure 3 shows how each search strategy performed in terms of the percent retrieved versus the total number of results returned. None of the search strategies that retrieved under 6 million results retrieved over 90% of the cited papers, and only one of the studies that retrieved over 6 million retrieved under 60% of the cited papers. Several search strategies achieved a higher percentage of retrieval, i.e., better precision, than search strategies that retrieved a considerably higher number of studies overall.

## Discussion

The overwhelming takeaway from this analysis is that there is little to no consistency in the way that review authors are searching for literature related to the SDOH. The sole exception is perhaps the database most frequently searched, given that all but two reviews included PubMed and/or Ovid MEDLINE. Beyond this, the investigation of review methods revealed a vast range of approaches, particularly among search strategies.

### Conceptual frameworks and definitions

While the World Health Organization’s CSDH framework stood out as the most-cited among the studied sample of reviews, there is not one single agreed-upon definition, model, or framework for the SDOH that is used to guide evidence syntheses conceptually. The literature supports our observation that the multiple SDOH models vary widely in their definitions of the determinants, as well as in the relationships between and among determinants and outcomes [35–37].

### Search strategies

There were over 20 reviews that searched PubMed and/or Ovid MEDLINE yet did not indicate searching for MeSH terms. Including controlled vocabulary terms has long been considered best practice for reviews [47–49]. While many of these reviews may well have captured the relevant MeSH terms by searching all available fields, using field codes to indicate which fields of the records are being searched is key for reproducibility and improves search precision [50, 51].

Among the searches that did incorporate controlled vocabulary, it was surprising to find that the MeSH term “social determinants of health” did not have a greater presence. Six of the reviews we analyzed reported conducting their searches before the MeSH term was introduced in 2014 and so would not have had it available to use for their search strategy. Among the 45 reviews that explicitly stated that their searches were conducted in or after the SDOH MeSH became available in 2014, over a third (*n* = 17, 37.78%) nevertheless omitted this seemingly essential term. Unfortunately, it was impossible to investigate this matter more precisely since 13 of the 64 reviews, or 20.3%, did not report the date that their searches were conducted, contrary to professional guidelines [50].

At the same time, even those reviews which use the SDOH MeSH term evidently cannot rely on this subject heading to retrieve much of the SDOH-related literature from MEDLINE, as only 61 (5.47%) of the 1115 cited papers indexed for MEDLINE between 2014 and 2022 were indexed with “social determinants of health.” Consequently, researchers undertaking evidence syntheses must incorporate additional terms to ensure broad retrieval of SDOH literature. This is increasingly crucial as databases move away from human indexing towards using automated processing for assigning controlled vocabulary terms. MEDLINE transitioned to an automated indexing process using the Medical Text Indexer (MTI) algorithm in 2022 [52] with mixed results [53, 54]. This points to the continued necessity of reflecting critically on the selection of keywords in addition to MeSH terms when drafting the search strategy for an evidence synthesis study. MeSH terms suggested as potentially relevant by the search strategies as well as by the indexing of the cited papers include Socioeconomic Factors, Risk Factors, Health Services Accessibility, Health Status Disparities, Healthcare Disparities, Poverty, and Educational Status. Further testing of the sensitivity and precision achieved with these terms will be necessary before making a final determination as to their usefulness.

No clear patterns emerged among the keywords selected by authors in their search strategies. Most reviews contained a mix of specific and general terms. We had hoped to analyze in detail which searches used specific and general terms and sort the terms into quantifiable categories. The constructs of *concept*/*measure* and *specific*/*non-specific terms* used by Prady and co-authors [32] also would have been interesting to explore. Unfortunately, these distinctions were not as easily quantified as first anticipated and ultimately beyond the scope of the project at hand. We did, however, observe that the ten SDOH search strategies with the highest number of search results—over 90% of cited papers—used a mix of both general and specific terms, and the ten strategies with the lowest number of search results used only notably general terms describing broad concepts, such as *determinants*, *disparities*, and *equities*. This implies potentially higher recall for a search that incorporates individual determinant terms as well as general concepts.

Notably, *risk factors* appeared frequently as both a MeSH term and keyword among the indexing of the cited papers, yet this term did not appear in any of the search strategies we analyzed. MeSH terms appearing frequently in both published search strategies and cited papers included Socioeconomic Factors, Health Status Disparities, Healthcare Disparities, and Health Services Accessibility. Keywords appearing frequently in both published search strategies and cited papers included *social determinants of health*, *social support*, *socioeconomic status*, and *race*. All of these MeSH terms and keywords were in the top 10 most frequently used among both published reviews and cited papers.

SDOH is a wide-ranging concept that can seem daunting, or even impossible, to comprehensively cover through a list of relevant search terms. Furthermore, SDOH frameworks often focus on broad domain definitions and fail to reflect adequate nuance [55]. When it came to translating their respective conceptualizations of the SDOH to search terms, some reviews analyzed in this study seemingly attempted to create exhaustive lists; others listed only a handful of terms. While most reviews cited at least one conceptual framework, only a small number of them made a clear connection between the framework and search strategy. No obvious recommendations emerged from the data in terms of a standard number of search terms to aim for.

### Recommendations for searching for SDOH literature

While the observations in the present study do not clearly lend themselves to the recommendation of any single search method, perhaps some guidance can be ascertained from the fact that the findings varied widely. Because there are so many ways to conceptualize the topic and myriad methods of searching, it seems of the highest importance that teams conducting evidence syntheses agree on a shared understanding of the SDOH concept. By selecting a framework or model to guide their review, authors can use a tangible definition of social determinants as a reference from which to derive search terms. Search strategies could be designed based on a particular framework, thus placing boundaries on the sprawling SDOH concept and giving authors a starting point while simultaneously providing clarity for the reader.

### Recommendations for authors writing about SDOH

Authors publishing original research related to the social determinants of health can use these findings to improve the discoverability of their abstracts by review teams and other searchers. Careful selection of the language used in article titles and abstracts may increase the possibility of the article being indexed with relevant terms. Notably, the MeSH term *social determinants of health* rarely appeared in article indexing unless that phrase also appeared in an article’s title or abstract.

### Limitations

The reviews analyzed in this study are a representative sample rather than an exhaustive list of all publications on the topic of SDOH. It is possible that the inclusion of reviews on other determinants (structural, commercial, etc.) would have had an impact on our findings, as would have related concepts such as health equity and disparities. Additionally, in 2020, there were significant changes made to the search function in PubMed [56]. PubMed searches executed at the time of our study may have had different results than at the time they were originally conceived and executed.

It is important to note that these findings reflect a sample collected at a moment in time; data were gathered in 2022 and analyzed between 2022 and 2023. Language used in academia and practice is constantly in flux and likely will continue to change as the field reflects and improves upon the way these concepts are described. For example, one recent publication encouraged researchers to reconceptualize population health and social determinants work in terms of *structural drivers* [57]*.* Ongoing observation of the language used to describe concepts related to SDOH and health equity is necessary for keeping search strategies current.

### Future research

The next phase of this research project will focus on the development and validation of a search hedge for identifying SDOH literature. The most commonly appearing MeSH terms and keywords from the reviews examined in this study, as well as the test set of cited papers, will be used as a foundation for constructing the hedge.

## Conclusions

While there has been considerable interest in the concept of SDOH, and many evidence syntheses include this as part of their research question, we found little consistency in terms of how researchers approach systematic searching for this concept. The differences in the terms and approaches used mean that the scope and quantity of the literature retrieved by these searches vary markedly. This has potentially significant implications for the overall amount of literature retrieved for evidence syntheses, and consequently incorporated into evidence-based public health policy and practice, when SDOH are a component. While MEDLINE was searched by nearly all evidence syntheses via PubMed and/or Ovid, and a MeSH term exists for SDOH, this subject heading was applied to only a small minority of the cited papers set, further highlighting the challenges in searching for SDOH literature.

A search hedge developed for SDOH could improve the recall of SDOH materials, bringing consistency and comprehensiveness to evidence syntheses. It remains unclear whether a precise and sensitive hedge will be achievable; however, this study has created a test set of SDOH literature that could potentially be used for that purpose. Until the time that such a hedge is developed and validated, researchers conducting evidence synthesis projects can improve transparency by choosing an SDOH framework to guide the selection of their search terms, thereby clarifying decisions. An expansive search strategy is likely necessary to recall all relevant evidence.

### Supplementary Information

Additional file 1.

## Data Availability

The dataset supporting the conclusions of this article is available in the University of Illinois Chicago repository, https://indigo.uic.edu/articles/dataset/Searching_for_the_social_determinants_of_health_dataset/24518497. The dataset was submitted to the University of Illinois Chicago repository on November 7, 2023, and accepted on November 13, 2023. An updated version was submitted and accepted on April 2, 2024.

## Abbreviations

DA: Date added
KW: Keywords
MeSH: Medical Subject Heading
PMID: PubMed Identification Number
SDOH: Social determinants of health
